# Uro-Vaxom® versus placebo for the prevention of recurrent symptomatic urinary tract infections in participants with chronic neurogenic bladder dysfunction: a randomised controlled feasibility study

**DOI:** 10.1186/s13063-019-3275-x

**Published:** 2019-04-16

**Authors:** Derick Wade, James Cooper, Fadel Derry, Julian Taylor

**Affiliations:** 1 Oxford Institute of Nursing, Midwifery and Allied Health Research (OxINMAHR), Faculty of Health & Life Sciences, Gipsy Lane Campus, Oxford, OX3 0BP, UK; 2 National Spinal Injuries Centre, Stoke Mandeville Hospital, Buckinghamshire Healthcare NHS Trust, Aylesbury, HP21 8AL UK; 3 Stoke Mandeville Spinal Research, National Spinal Injuries Centre, Aylesbury, HP218 AL UK; 4 Harris Manchester College, University of Oxford, Oxford, OX1 3TD UK; 5 Sensorimotor Function Group, Hospital Nacional de Parapléjicos, SESCAM, Finca ‘La Peraleda’, 45071 Toledo, Spain

**Keywords:** Symptomatic urinary tract infection, *Escherichia coli*, Prophylactic immunotherapy, Uro-Vaxom®, Lower neurogenic bladder dysfunction, Spinal cord injury, Multiple sclerosis, Transverse myelitis, Cauda equina syndrome

## Abstract

**Background:**

Patients with lower neurogenic bladder dysfunction are at an increased risk of suffering from recurrent urinary tract infections. Recurrent symptomatic urinary tract infection is occasionally treated with antibiotics as a prophylactic prevention strategy. This risks increasing the frequency of antibiotic resistance. National healthcare policymakers have requested further research into alternative preventive measures for pathologies that require antibiotic treatment.

**Methods:**

This study protocol describes a two-centre, randomised, double-blinded, placebo-controlled study to evaluate the prevention of recurrent urinary tract infections with the commercial immunotherapy agent Uro-Vaxom®, based on *Escherichia coli* pathogen-associated molecular patterns. Eligible participants are recruited by the direct healthcare team and randomised to receive Uro-Vaxom® in the form of an oral capsule, or a matching placebo. Participants will receive the study treatment daily for 3 months and followed up for an additional 3 months so that the number of symptomatic urinary tract infection episodes and individual signs and symptoms per episode can be recorded using participant study diaries. Primary outcome measures are: number of symptomatic urinary tract infections experienced over 3 months, number of symptomatic urinary tract infections experienced over 6 months, time from the start of treatment to the first urinary tract infection, and the presence of asymptomatic bacteriuria at 3 and 6 months. Secondary outcome measures are: individually recorded symptoms normally associated with recurrent urinary tract infection and consistency of reported symptoms during the symptomatic urinary tract infection experienced during the study, compliance with study protocol and study medication, and adverse events.

**Discussion:**

Healthcare policymakers recommend that alternative preventative strategies are identified for symptomatic urinary tract infections that require antibiotic treatment. If Uro-Vaxom® is shown to be effective, this feasibility study would warrant a larger, statistically powered, multicentre study to investigate whether this immunotherapy strategy is an effective preventative measure for recurrent symptomatic urinary tract infection for people with spinal cord injuries and neurological pathologies.

**Trial registration:**

ISRTCN. Registered on 30 October 2015.

ClinicalTrials.gov, ID: NCT0251901. Registered on 30 October 2015.

URL of trial registry record: Ethics Ref: 15-LO-2069. IRAS Number: 185760. Sponsor Number: RXQ/648.

NIHR Funding Reference: PB-PG-1013-32017.

**Electronic supplementary material:**

The online version of this article (10.1186/s13063-019-3275-x) contains supplementary material, which is available to authorized users.

## Background

Lower neurogenic bladder dysfunction as a result of brain, spinal cord or nerve damage often leads to enhanced risk of symptomatic urinary tract infections (UTIs), due to defective urine storage and voiding, bladder stones, foreign bodies or residual urine in the bladder [1]. Symptomatic UTIs increase healthcare costs [2] by exacerbating problems of incontinence, contributing to poor neurological bladder control, more cases of hospital admission, and potentially leading to reduced renal function which in some cases is life-threatening. Patients with neurogenic bladder dysfunction may experience several symptomatic UTI episodes each year [3], with the bacteria *Escherichia coli* (E Coli), responsible for approximately 80% these events [4]. Common specific causes of neurogenic bladder dysfunction are spinal cord injury (SCI), cauda equina syndrome, multiple sclerosis or transverse myelitis [5, 6].

Research on UTIs in participants with neurogenic bladder dysfunction is faced with methodological difficulties [7]. There are checklists for UTI symptoms and signs available from National Institute for Health and Care Excellence (NICE) for men and women with uncomplicated UTIs [8, 9], where no functional or structural abnormalities of the urinary tract are detected. However, these checklists have limited value in patients with neurogenic bladder dysfunction. Given that these groups of patients normally do not feel symptoms like ‘pain’ and ‘a frequent urge to urinate’, it can be difficult to distinguish asymptomatic bacteriuria and a symptomatic UTI. To date, there is no clear agreement among experts on which signs and symptoms are indicative of a symptomatic UTI in participants with neurogenic bladder dysfunction.

The first line of treatment for symptomatic UTIs includes prescription of antibiotics [7]. If the infections are recurrent, patients may be prescribed antibiotics as a prophylactic treatment. The effectiveness of this strategy is unknown; however, it is not recommended as it encourages the development of multi-drug-resistant bacteria [1].

Research into alternative, preventative treatments for people with recurrent symptomatic UTIs has been recommended [7]. Research into the prevention of symptomatic UTIs in people with Spinal Cord Injury (SCI) has centred around effective methods for bladder drainage [10], nutritional supplements, such as cranberries [11], or prophylactic antibiotic treatment [1]. One possible preventative treatment strategy is the use of immunotherapy agents [12], such as Uro-Vaxom®, which consists of bacterial lysates from E Coli that mediate its effect by the ability of bacterial component pathogen-associated molecular patterns (PAMP) to non-specifically stimulate cells of the innate immune system [13].

Most clinical studies of Uro-Vaxom® have been performed in able-bodied individuals or people with SCI who suffer from recurrent UTIs. These studies have shown that the treatment is well tolerated, and more effective than placebo in reducing the number of events of UTI [14–17]. Furthermore, in one prospective trial of 70 people with SCI, the effectiveness of Uro-Vaxom® was demonstrated to reduce symptomatic UTI events, which persisted and even increased over time [18]. A retrospective review of data from people with SCI [10] and a retrospective cohort study using people with SCI gives weak supportive evidence of a positive effect with Uro-Vaxom® for UTI [19].

This study will investigate whether Uro-Vaxom® reduces the incidence of symptomatic UTIs in people with neurogenic bladder dysfunction due to SCI, cauda equina syndrome, transverse myelitis or multiple sclerosis. The study design is based on a randomised, double-blind, placebo-controlled, parallel-group, 1:1 design to compare active Uro-Vaxom® immunotherapy over 3 months compared against an inactive matching placebo. Clinical outcome measures will be recorded at 12 weeks and at 26 weeks (3 months following the end of treatment). The overall objective of the feasibility study is to collect sufficient pilot data to design a larger, statistically powered, multicentre trial in the UK.

The specific objectives are to:

- Establish the feasibility of:
  - ◦ recruiting patients at different sites
  - ◦ retaining participants in the study
  - ◦ collecting data from questionnaires and urine samples
- Investigate the benefit of Uro-Vaxom® for:
  - ◦ reduction of symptomatic infections treated with antibiotics
  - ◦ reduction of asymptomatic bacteriuria at 3 and 6 months
- Investigate the consistency of the participant’s specific symptoms associated with infection by:
  - ◦ recording individual characteristic UTI symptoms recorded at baseline for each participant with those symptoms normally experienced during a symptomatic UTI
  - ◦ recording which of the baseline individual characteristic symptoms are experienced with symptomatic UTI during the study
- Monitor adverse events associated with taking active Uro-Vaxom®
- Plan an extended multicentre, Phase III study using collected data to:
  - ◦ calculate the number of participants needed for a statistically powered trial
  - ◦ assess sensitivity of outcome measures to detect prevention of symptomatic UTI

This study is funded by the UK National Institute for Health Research (NIHR) through its Research for Patient Benefit (RfPB) funding stream, reference number PB-PG-1013-32017 with the title ‘Prevention of Recurrent Symptomatic Urinary Tract Infections in patients with chronic Neurogenic Bladder dysfunction: a mixed method study (The PReSUTINeB Study)’. The active and placebo drug packs are provided by the manufacturing company OM Pharma, a partner company of Vifor Pharma Group, Switzerland.

## Methods

### Study history

The original protocol was registered at ClinicalTrials.Gov in October 2015 (NCT02591901) and the first part of the study, unrelated to the interventional phase administration of Uro-Vaxom®, began in April 2016. The Study Steering Group was set up in January 2017 and recommended changes in the original, funded protocol to improve the primary and secondary outcome measures of the interventional phase. In addition, the Study Steering Group recommended collection of UTI symptoms characteristic for each individual participant. The new version of the protocol was developed in accordance to the Standard Protocol Items: Recommendations for Interventional Trials (SPIRIT) checklist [Additional file 1].

### Study design

The study is a prospective, randomised, double-blinded, placebo-controlled study of 48 adults who meet the inclusion criteria. Participants will be on the study treatment or placebo for 3 months and followed up for 3 months after.

### Setting

The study is embedded within the National Health Service in the UK; this service is responsible for delivering the great majority of healthcare to the population, especially to people with long-term disabling conditions. The two centres within this study are Stoke Mandeville Hospital (part of Buckinghamshire Healthcare NHS Trust) and Oxford Centre for Enablement (part of Oxford University Hospitals NHS Foundation Trust).

### Study aims

#### Primary aim

To investigate if Uro-Vaxom® can reduce the incidence of symptomatic UTIs that require antibiotic treatment in patients who suffer with recurrent UTIs as a result of a SCI, cauda equina syndrome, transverse myelitis or multiple sclerosis.

#### Secondary aim

To investigate the symptoms associated with UTIs that require antibiotic treatment, and how consistently they are reported in an individual participant.

### Study sample

#### Eligibility criteria

Eligibility criteria include; clinical diagnosis of SCI, multiple sclerosis, transverse myelitis or cauda equina syndrome; diagnosis of the spinal pathology for at least the previous 12 months; no significant changes in the underlying condition for 12 weeks; living in the community (not in residential care); aged 18 to 75 years; have had at least three UTI episodes treated using antibiotics over the preceding 12 months; if a woman of child-bearing age, is willing to use contraception for the duration of the study; and having the mental capacity to give informed consent.

Patients will be excluded if they: have had surgical alterations to the bladder, excluding supra-pubic catheterisation; have known hypersensitivity to the active principle or to any of the excipients of Uro-Vaxom® or are unwilling to take a product containing bovine gelatin (e.g. vegetarians).

#### Sample recruitment procedures

Patients will be recruited either from: patients attending or contacting specialist neurological and/or rehabilitation services, as outpatients or day-patients but also through the out-reach programme; through non-statutory patient support organisations, using newsletters and websites, advertisements organised from participating sites using all standard and social media outlets.

Patients who are interested in participating in the study will be given an information leaflet in person or sent one in the post. If the patient is interested and considers themselves appropriate for the study, they will be interviewed at one of the study sites as an outpatient. The patient will meet a physician or nurse and at this meeting, once the eligibility is confirmed, informed consent will be signed by the patient before any formal clinical data are collected. The current version of the informed consent form is at the end of this document [Additional file 2].

Baseline information will then be collected. These data will include: demographic data including spinal pathology diagnosis, year of onset of underlying condition, gender and age at time of enrolment; symptoms that the individual participant associates with having a UTI that requires antibiotic treatment; and a cultured urine sample. Note that the presence of asymptomatic bacteriuria will not render the patient ineligible.

If the participant is already on antibiotic treatment and is eligible to take part in the study, the participant can be enrolled but will be advised not to start the study treatment until after the course of antibiotics has finished and until at least 14 days have passed from the last UTI symptoms.

Once baseline data are collected and subject registration tasks have been completed, including obtaining informed consent, the participant will return home.

### Randomisation and blinding

Eligible patients who have given their consent will be registered for the study by their physician or nurse, and then randomly allocated (1:1) to one arm of the study using a centralised and remote computer-based allocation randomisation system, Registration/Randomisation and Management of Product (RRAMP), provided by the Oxford Clinical Trials Research Unit (OCTRU) based on a non-deterministic minimisation algorithm, to ensure treatment concealment and balanced allocation of participants across the two treatment groups.

Following group allocation, the randomisation service will inform the local pharmacy and the local research team which numbered treatment pack to use for each participant; the pharmacy, research team or the participant will not know which treatment it contains. The drug pack will be dispensed by the hospital pharmacy to an appropriate research team before being given to the participant with instructions to take one capsule every day during the treatment phase (Day 1 to the last day of Month 3). Each capsule will be taken orally in the morning before breakfast.

During the study the participant may start or stop any treatment, including those taken in relation to their neurological condition and any other treatment (which includes antibiotics for treatment of symptomatic UTI). The treating physician will provide guidance regarding treatments taken during the study. No treatment will be withheld during the study.

### Baseline assessment (Day 0)

The participant will answer baseline questions on bladder management, current medication (including antibiotic use for symptomatic UTI), number of symptomatic UTIs experienced in the previous 12 months and the use of ‘rescue antibiotics’ (antibiotics stored and used when experiencing symptoms of suspected UTI before or without visiting to see a general practitioner for confirmation). The presence of catherisation (both intermittent and indwelling) will be recorded.

### Intervention

The active intervention, Uro-Vaxom®, will be presented as yellow/orange capsules prepared for oral administration. The active treatment contains 6 mg active lyophilised bacterial lysate from 18 E Coli strains per capsule and 54 mg mannitol as a loading agent. The inactive placebo intervention is matched with the same excipients; pre-gelatinised modified maize starch, magnesium stearate, propyl gallate (E 310), sodium glutamate, mannitol, bovine gelatin, ferric and titanium dioxide.

The participants will be asked to return any unused capsules at the follow-up assessment at 12 weeks; this will allow calculation of maximum compliance. There is no additional procedure to confirm actual compliance for taking capsules.

### Concealment and emergency unblinding

Adverse events reported spontaneously, or at the regular reviews at 4, 12 and 26 weeks, will be recorded. If the adverse event is assessed to be directly attributed to the treatment, and if the adverse event is serious, non-trivial or short-lived, then the possibility of stopping treatment will be considered. In the unlikely event of an adverse event occurring that requires knowledge of the participant’s allocated group, the allocation code will be broken (see below).

### Outcomes (see Fig. 1)

The outcome measures and the time at which they are recorded are shown in Fig. 2.

**Fig. 1:**
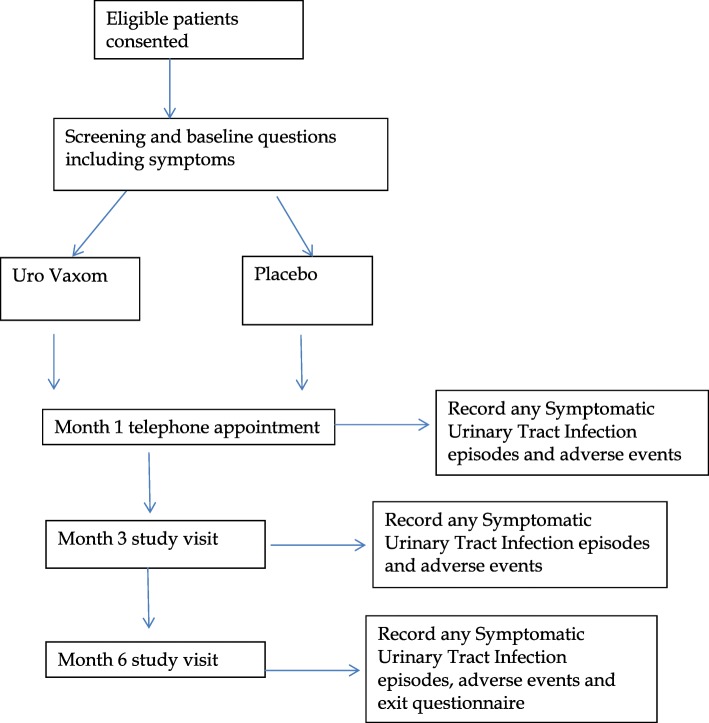
Consolidated Standards of Reporting Trials (CONSORT) Diagram

**Fig. 2:**
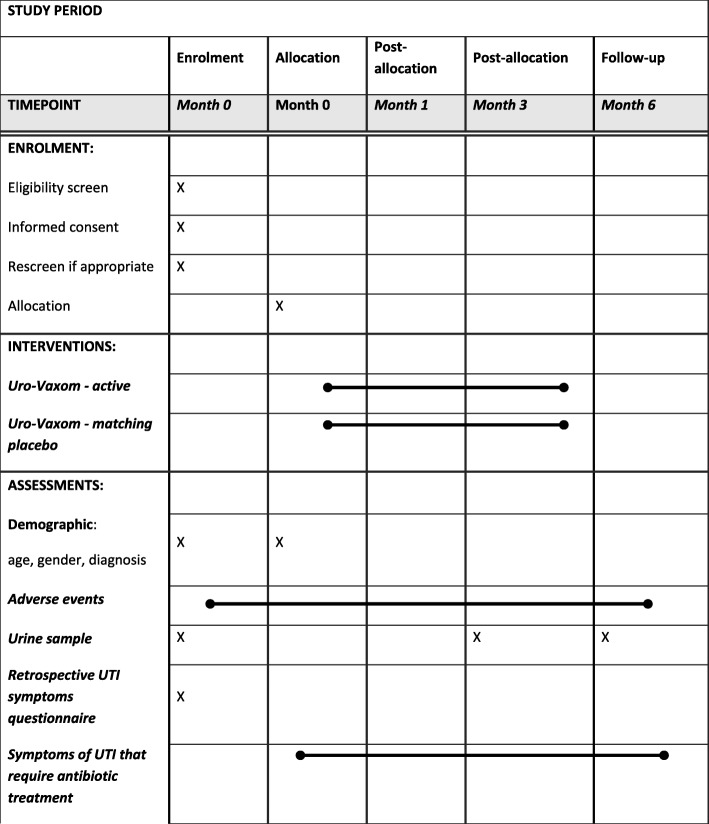
Standard Protocol Items: Recommendations for Interventional Trials (SPIRIT) Diagram

The primary clinical outcome measures are:

- number of symptomatic UTIs experienced over the first 3 months
- number of symptomatic UTIs experienced over the second 3 months
- time from start of treatment to the first symptomatic UTI
- the presence of asymptomatic bacteriuria at 3and 6 months

The secondary clinical outcome measures are:

- symptoms associated with UTIs, and how consistently they are reported by an individual participant
- compliance with:
  - ◦ protocol, e.g. attending follow-up and completing questionnaires
  - ◦ taking Uro-Vaxom® in oral capsule form
- adverse events (including serious adverse events)

A UTI event is defined as thus; when the participant develops symptoms suggesting a UTI and they act as normal to seek medical attention; usually this will be to consult with their physician. If the physician diagnoses a UTI and prescribes an antibiotic for this infection, the participant will be considered to have had a clinically diagnosed UTI. It is understood that participants may have ‘rescue packs’ of antibiotics at home to take in the event of a suspected UTI without having to go to the physician. It will be presumed that a UTI has also occurred if a participant starts to take a rescue pack of antibiotics.

The research team will not undertake any specific investigation nor will it be involved in any treatment or other management decisions. However, the team will ask the participant to complete a form recording the symptoms experienced by completing a checklist of symptoms normally experienced during any UTI episode. The form will be collected from the participant and the name and dose of the antibiotic prescribed will be recorded. If the participant has further UTI episodes, the same procedures will occur and the signs and symptoms will be recorded in a diary that the participants take home with them.

### Adverse events

The study will record all adverse events in the Case Report Form. An adverse event is defined as ‘any untoward medical occurrence occurring from the moment the participant signs the Informed Consent Form until the participant exits the study. It does not need to have a causal relationship with the study drug or placebo’. An adverse reaction is defined as ‘any adverse event which has any reasonable possibility it was caused by study drug or placebo’ [20]. Participants will be asked about these at each review or other contact and will be asked to report any adverse event experienced.

The adverse event or reaction for this study will be classified as serious if it: results in death; is life-threatening; requires hospitalisation or prolongation of existing hospitalisation; results in persistent or significant disability or incapacity [20]; or is a significant medical event in the investigator’s judgement.

If a serious adverse event occurs, the participant or treating clinician will need to contact their local study contact immediately. The site investigator or delegate will immediately complete the Serious Adverse Event Form, especially the likelihood of being related to the investigated product or placebo and notify the Study Management Group which will determine what further actions might be needed.

A suspected unexpected serious adverse reaction is a serious adverse reaction to the study drug or placebo in which the nature or severity is not consistent with the applicable product information (e.g. reference safety information). All serious adverse events will be reported to the Research Ethics Committee and the sponsor within 15 days of the chief investigator becoming aware of the event.

### Research ethics and governance

The original protocol was registered at ClinicalTrials.gov at the end of October 2015 in collaboration with the OCTRU, reference number NCT02591901. The specific ethical review took place at its London-Harrow Research Ethics Committee (ref: 15/LO/2069), and the study received a favourable opinion on 1 March 2016 The sponsor is Buckinghamshire Healthcare NHS Trust, and the contact is Ms Denise Watson (denise.watson4@nhs.net).

**Fig. 3:**
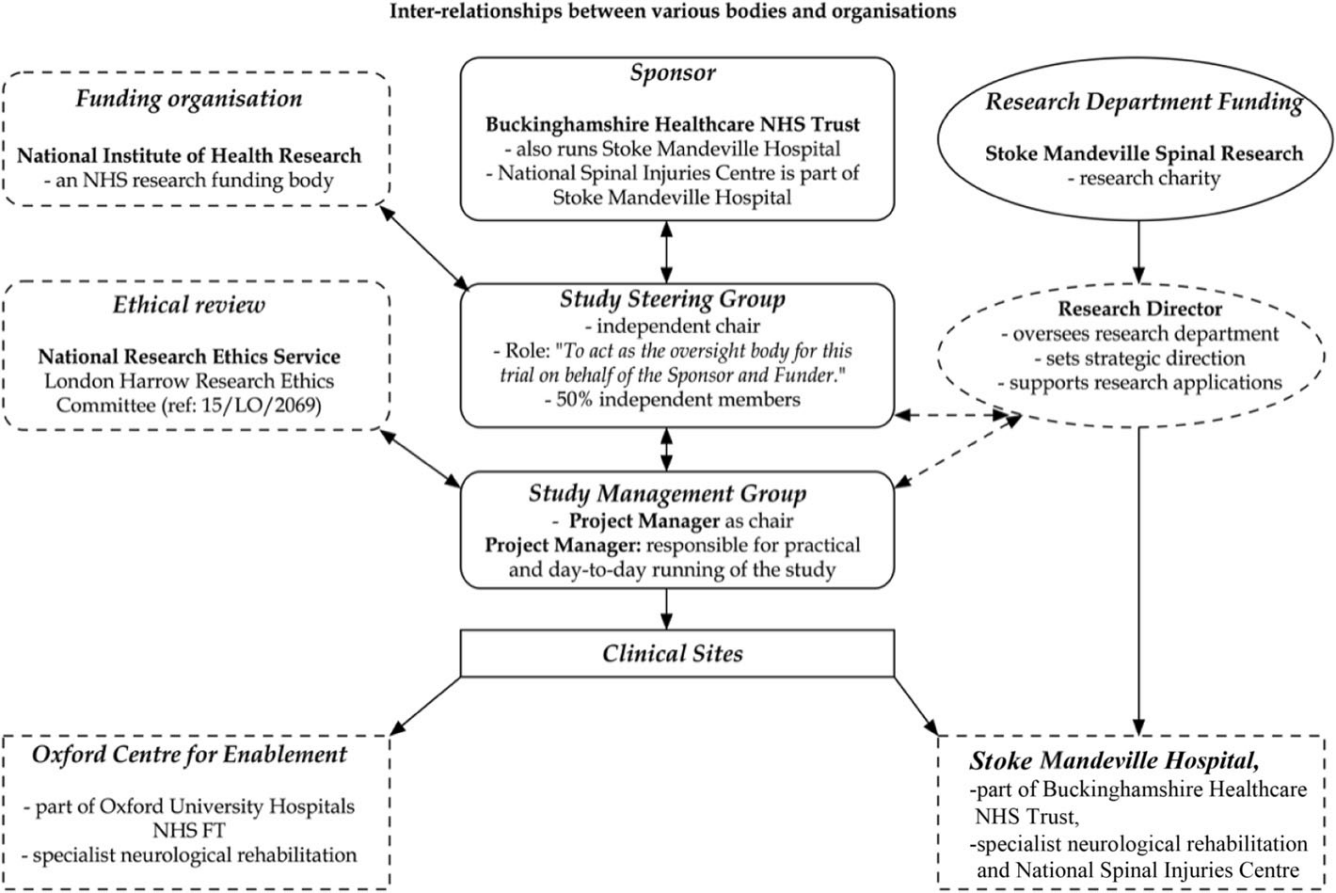
Inter-relationship between sponsor, funding, research governance, study management and clinical research sites

The study sponsor and the funding body have had, and will have, no active influence over the design of the study since it obtained funding, and does not, and will not, have any influence over the project’s management, the collection and handling of data, writing up the papers and reports that are generated, or decisions on what to publish or where. The study sponsor will be informed of the study progress. The Study Steering Group will provide oversight to the study on behalf of the sponsor and the Study Management Group will be responsible for daily management. There is no Data Monitoring Committee because the study was designed as a feasibility study on a limited number of participants, and is not designed for statistical power. The inter-relationship structure is shown in Fig. 3.

### Sample size

No estimate of sample size is needed for the primary outcome measures or for the expected effect size of any treatment, as the study is a feasibility study to develop methodological processes and procedures required for a larger, statistically powered study. The recently published revision of the Consolidated Standards of Reporting Trials (CONSORT) Statement for feasibility studies [21] explains why a sample size should be calculated, and how, but at the time that this study protocol was first written (2015) it was not subject to such guidance. The study will recruit a total of 48 participants. This is sufficient to justify a larger study if the effect size is medium (0.5).

### Data management

All primary source data will be recorded on prepared paper case report forms, checked by the principal investigator, signed off by the principal investigator or delegate and then entered into a secure electronic database run by the OCTRU. The paper records will be stored securely in locked file cabinets in a secure area under lock and key. Participant details and study forms will be forwarded to the central study office based in Stoke Mandeville Hospital. These will be securely stored and used to coordinate the follow-up and other study processes as appropriate. The electronic database will use the participant’s randomisation number to identify participants. Once all data are collected, the electronic data files will be checked, validated and locked.

Privacy and confidentiality of the participant’s medical and study data will be maintained through the study. The file containing the participant identifier codes and all case report forms will be kept in locked file cabinets in secure areas at the NHS Trust premises and will only be accessible to the local investigator and study coordinator. The OCTRU’s electronic data capture system is also located at a secure premise, includes restricted access and maintains a continuous advanced mirroring back-up system on secure operating servers.

NHS-employed health professionals involved in this study are expected to adhere to the ‘NHS Codes of Practice’, in particular the ‘Code of Confidentiality’.

### Data analysis

Primary analysis comparing the two intervention groups will be undertaken blinded, without knowing which group has taken active medication. A senior statistician from the OCTRU developed the statistical methods for the study. The primary statistical analysis will be carried out on the basis of intention-to-treat, with all participants being analysed according to their allocated treatment group irrespective of which treatment they actually receive.

As this is a feasibility study and is not statistically powered for hypothesis testing, analysis will be descriptive in nature and will focus on confidence interval estimation in order to provide key data parameters for the larger, definitive trial. The principle analysis will compare results between Uro-Vaxom® and placebo groups.

As part of the Statistical Analysis Plan, feasibility parameters will include measures that summarise response, eligibility, consent and randomisation rates and will be presented as point estimates with 95% confidence intervals. Secondary analysis will provide the same rates adjusted for method of bladder management (catheter or no catheter use) and centre. The number of non-compliant participants and attrition (from treatment, follow-up or both) in each arm will be reported. The extent of missing data will also inform the feasibility of selected measurement tools. Data will be analysed once complete data collection, entry and validation is achieved.

## Discussion

This study will explore a new approach for the prevention of recurrent symptomatic UTIs in people with neurogenic bladder dysfunction as a result of SCI, cauda equina syndrome, transverse myelitis or multiple sclerosis, using the immunotherapy Uro-Vaxom®. The majority of trials using Uro-Vaxom® have shown an effect in reducing UTI events in able-bodied individuals [14–17] or participants with SCI [18].

One of the study’s primary outcome measures, the occurrence of a symptomatic UTI requiring antibiotic treatment, is clinically valid and is relevant to the patient. However, the weakness of this measure is the subjective nature of how the treating clinician defines symptomatic UTI, with both the patient and the clinician relying upon past experience and identification of individual specific symptoms. It is important to note that this study will not base a clinical diagnosis on analysis of microbial urine. Furthermore, a study by Sundén et al. [22] found that the presence of asymptomatic bacteriuria could be correlated with a positive outcome (reduction of UTI recurrences).

The decision to define the primary outcome measure, based on the prescription of an antibiotic for symptomatic UTI, reflects the clinical reality where currently no external validation of diagnosis is possible [7].

The subjective nature of the primary outcome measure based on the diagnosis of a UTI event is balanced by the objective secondary outcomes that will quantify a reduction in asymptomatic bacteriuria during 3 and 6 months after treatment initiation. An early study investigating Uro-Vaxom® recorded a progressive reduction in asymptomatic bacteriuria in the active treatment group compared to the control group [18]. A reduction in bacterial count with oral Uro-Vaxom® will provide strong independent evidence of its effectiveness for people with infections with neurogenic bladder dysfunction. However, it is also acknowledged that asymptomatic infection with E Coli might actually protect against symptomatic UTI [22].

A practical concern related to the inclusion criterion is limiting recruitment to patients who have experienced three or more infection episodes over a period of 12 months. Although the recruitment of patients with high rates of recurrent UTI may limited total numbers recruited, it may be that the effect will be more easily detected, assuming (without evidence) that the past rate of recurrent infection predicts the future rate. If this study shows potential benefit for reduced UTI events, the larger study will be designed to recruit patients with lower rates of UTI which will be more in line with clinical need. A larger trial will also be designed to account for other factors that may influence symptomatic UTI development such as gender or age.

The diversity of this patient population, including possible differences in UTI symptoms between the different pathologies and possible differences in a participant between different episodes, presents difficulties with regard to the definition of a symptomatic UTI for patients with neurogenic bladder dysfunction. Due to the absence of a consensus for the definition of symptomatic UTI in patients with neurogenic bladder dysfunction, this study will ask participants to record their own individual signs and symptoms experienced normally during symptomatic UTIs that require antibiotic treatment, at baseline, using free text and experienced at every symptomatic UTI episode (tick box from list) during their time on the study. Also, during this time participants will also be asked if the antibiotic treatment (either prescribed or rescue antibiotics) rendered the participant symptom free. This will add to the knowledge base and may assist in establishing a better means for diagnosis, although, due to the limitations in diagnosis and prescription discussed previously, this objective may be limited.

This study will not withhold or alter routine treatment or care for the purpose of this study. Any new or modifications to current medication will be documented at each participant contact after enrolment. The study team had chosen to include patients currently taking prophylactic antibiotics and to not withdraw participants if they prescribed such preventative treatment during their time on the study.

### Study status

The current approved protocol at time of publication is version 9.0 dated 9 May 2018. Recruitment for the survey and interviews (previously named stage I, now terminated) began on 28 March 2016. Recruitment for the intervention began on 13 April 2018 and it will be completed before 21 April 2019.

## Additional files

Additional file 1: Standard Protocol Items: Recommendations for Interventional Trials (SPIRIT) 2013 Checklist: recommended items to address in a clinical trial protocol and related documents*. (DOC 120 kb)

Additional file 2: Informed consent materials. (DOCX 22 kb)

## Abbreviations

CONSORT: Consolidated Standards of Reporting Trials
E Coli: *Escherichia coli*
OCTRU: Oxford Clinical Trials Research Unit
PReSUTINeB: Prevention of Recurrent Symptomatic Urinary Tract Infections in patients with chronic Neurogenic Bladder dysfunction: a mixed method study
SCI: Spinal cord injury
UTI: Urinary tract infection

## References

[1] Salomon J, Denys P, Merle C, Chartier-Kastler E, Perronne C, Gaillard JL, Bernard L (2006). Prevention of urinary tract infection in spinal cord-injured patients: safety and efficacy of a weekly oral cyclic antibiotic (WOCA) programme with a 2-year follow-up—an observational prospective study. J Antimicrob Chemother.

[2] Manack A, Motsko SP, Haag-Molkenteller C, Dmochowski RR, Goehring EL, Nguyen-Khoa BA, Jones JK (2011). Epidemiology and healthcare utilization of neurogenic bladder patients in a US claims database. Neurourol Urodyn.

[3] Esclarin De Ruz A, Garcia Leoni E, Herruzo Cabrera R (2000). Epidemiology and risk factors for urinary tract infection in patients with spinal cord injury. J Urol.

[4] Khatri B, Basnyat S, Karki A, Poudel A, Shrestha B (2012). Etiology and antimicrobial susceptibility pattern of bacterial pathogens from urinary tract infection. Nepal Med Coll J.

[5] Ginsberg D (2013). The epidemiology and pathophysiology of neurogenic bladder. Am J Manag Care.

[6] D'Hondt F, Everaert K (2011). Urinary tract infections in patients with spinal cord injuries. Curr Infect Dis Rep.

[7] National Institute for Healthcare and Clinical Excellence: Urinary incontinence in neurological disease: assessment and management. https://www.nice.org.uk/guidance/cg148. Accessed 23 Sept 2018. 31869051

[8] National Institute for Healthcare and Clinical Excellence: Urinary tract infection (lower) – women. 2009. http://cks.nice.org.uk/urinary-tract-infection-lower-women. Accessed 23 Sept 2018.

[9] National Institute for Healthcare and Clinical Excellence: Urinary tract infection (lower) – Men. 2010. https://www.nice.org.uk/guidance/cg97. Accessed 23 Sept 2018.

[10] Salameh A, Al Mohajer M, Daroucihe RO (2015). Prevention of urinary tract infections in patients with spinal cord injury. CMAJ.

[11] Hess MJ, Hess PE, Sullivan MR, Nee M, Yalla SV (2008). Evaluation of cranberry tablets for the prevention of urinary tract infections in spinal cord injured patients with neurogenic bladder. Spinal Cord.

[12] Barclay J, Veeratterapillay R, Harding C (2017). Non-antibiotic options for recurrent urinary tract infections in women. BMJ.

[13] Huber M, Ayoub M, Pfannes SD, Mittenbuhler K, Weis K, Bessler WG, Baier W (2000). Immunostimulatory activity of the bacterial extract OM-8. Eur J Med Res.

[14] Bauer HW, Alloussi S, Egger G, Blumlein HM, Cozma G, Schulman CC (2005). Multicenter UTISG.:A long-term, multicenter, double-blind study of an *Escherichia coli* extract (OM-89) in female patients with recurrent urinary tract infections. Eur Urol.

[15] Schulman CC, Corbusier A, Michiels H, Taenzer HJ (1993). Oral immunotherapy of recurrent urinary tract infections: a double-blind placebo-controlled multicenter study. J Urol.

[16] Magasi P, Panovics J, Illes A, Nagy M (1994). Uro-Vaxom and the management of recurrent urinary tract infection in adults: a randomized multicenter double-blind trial. Eur Urol.

[17] Tammen H (1990). Immunobiotherapy with Uro-Vaxom in recurrent urinary tract infection. The German Urinary Tract Infection Study Group. Br J Urol.

[18] Hachen HJ (1990). Oral immunotherapy in paraplegic patients with chronic urinary tract infections: a double-blind, placebo-controlled trial. J Urol.

[19] Krebs J, Fleischli S, Stoyanov J, Pannek J (2019). Effects of oral immunomodulation therapy on urinary tract infections in individuals with chronic spinal cord injury—A retrospective cohort study. Neurourol Urodyn.

[20] International Council for Harmonisation of Technical Requirements for Pharmaceuticals for Human Use (ICH): Medicines for Human Use (Clinical Trials) Regulations. 2004. http://www.legislation.gov.uk/uksi/2004/1031/made. Accessed 23 Sept 2018.

[21] Eldridge SM, Chan CL, Campbell MJ, Bond CM, Hopewell S, Thabane L, Lancaster GA, PAFS Consensus Group (2016). CONSORT 2010 Statement: extension to randomised pilot and feasibility trials. BMJ.

[22] Sunden F, Hakansson L, Ljunggren E, Wullt B (2010). *Escherichia coli* 83972 bacteriuria protects against recurrent lower urinary tract infections in patients with incomplete bladder emptying. J. Urol.

