# Supplementary material for: Uro-Vaxom® versus placebo for the prevention of recurrent symptomatic urinary tract infections in participants with chronic neurogenic bladder dysfunction: a randomised controlled feasibility study
Source: Trials. 2019 Apr 16;20:223. doi: 10.1186/s13063-019-3275-x (PMC6469220; doi:10.1186/s13063-019-3275-x)
Supplement: Supplementary file 2 — Informed consent materials. (DOCX 22 kb) [file 13063_2019_3275_MOESM2_ESM.docx]

**Additional file 2: Informed consent materials**

**INFORMED CONSENT FORM**

**Short study title: PReSUTINeB study**

**Research Ethics Committee reference: 15-LO-2069**

**IRAS reference: 185760**

| \| **Condition** \| **(Patient) Please initial box** \| \| --- \| --- \| \| 1. I confirm I have read and understand the information sheet dated .................... for the above study. I have had the opportunity to consider the information, ask questions to a member of the research team and have had these answered satisfactorily. \|  \| \| 1. I understand that my participation is voluntary and that I am free to withdraw at any time without giving any reason. \|  \| \| 1. I understand that relevant sections of my data collected during the study, may be looked at by appropriate individuals from the research team, NHS Trust, Research Ethics Committee or Regulatory Authority, where it is relevant to my taking part in this research. I give permission for these individuals to have access to my medical records and study data. \|  \| \| 1. I understand that any urine sample provided during this study will be analysed for safety monitoring reasons and for recording information relevant to the results of the above study. Urine samples will only be examined during this study and will be destroyed at the end of the study. \|  \| \| 1. I agree that my GP will be informed about my participation in the study.   [*Note: Not agreeing with this does NOT mean that the*  *participant cannot participate*] \|  \| \| 1. I agree to allow my details and any completed forms to be forwarded to the PReSUTINeB study office at Stoke Mandeville Hospital and securely stored for the purposes of the PReSUTINeB study only. \|  \| \| 1. I understand that one product contains gelatine that is sourced from cows. \|  \|  \| **Condition** \| **(Patient) Please initial box** \| \| --- \| --- \| \| 1. I understand that if I am enrolled whilst taking antibiotics for a urinary tract infection, I must not take the study drug until 14 days after I have stopped taking the antibiotics and symptoms have subsided. I understand that I will confirm with the local study team when I start taking the study drug. \|  \| \| 1. I agree to take part in the above named study. \|  \| | | |
| --- | --- | --- | --- | --- | --- | --- | --- | --- | --- | --- | --- | --- | --- | --- | --- | --- | --- | --- | --- | --- | --- | --- | --- | --- |
| ______________________ | _______________________ | ________________________ |
| Participant Name | Date | Signature |
| *If participant is unable to give written consent and has given verbal consent to the specific items as described and marked above.* | | |
| _______________________ | _______________________ | _______________________ |
| Witness Name (Relationship) | Date | Signature |

| 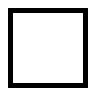 **Please initial box**     1. I have explained all the relevant aspects of the research to the participant and answered their questions. I have pointed out that participation in the research project is completely voluntary and that they may stop their participation at any time. | | |
| --- | --- | --- |
| _______________________ | _______________________ | _______________________ |
| Individual Obtaining Consent Name | Date | Signature |

*When completed: 1 (original) to be kept in research record, 1 for participant; 1 for researcher site file.*
